# Changing hygiene behaviours: a cluster-randomized trial, Ethiopia

**DOI:** 10.2471/BLT.21.285915

**Published:** 2021-08-30

**Authors:** Solomon Aragie, Wondyifraw Tadesse, Adane Dagnew, Dagnachew Hailu, Melese Dubie, Dionna M Wittberg, Jason S Melo, Mahteme Haile, Taye Zeru, Matthew C Freeman, Scott D Nash, E Kelly Callahan, Zerihun Tadesse, Benjamin F Arnold, Travis C Porco, Thomas M Lietman, Jeremy D Keenan

**Affiliations:** a The Carter Center Ethiopia, Addis Ababa, Ethiopia.; b Catholic Relief Services, Addis Ababa, Ethiopia.; c Francis I Proctor Foundation, University of California San Francisco, 490 Illinois Street, Floor 2, Box 0944, San Francisco, CA 94158, United States of America (USA).; d Amhara Public Health Institute, Bahir Dar, Ethiopia.; e Rollins School of Public Health, Emory University, Atlanta, USA.; f The Carter Center, Atlanta, USA.

## Abstract

**Objective:**

To determine whether a water, sanitation and hygiene intervention could change hygiene behaviours thought to be important for trachoma control.

**Methods:**

We conducted a cluster-randomized trial in rural Ethiopia from 9 November 2015 to 5 March 2019. We randomized 20 clusters to an intervention consisting of water and sanitation infrastructure and hygiene promotion and 20 clusters to no intervention. All intervention clusters received a primary-school hygiene curriculum, community water point, household wash station, household soap and home visits from hygiene promotion workers. We assessed intervention fidelity through annual household surveys.

**Findings:**

Over the 3 years, more wash stations, soap and latrines were seen at households in the intervention clusters than the control clusters: risk difference 47 percentage points (95% confidence interval, CI: 41–53) for wash stations, 18 percentage points (95% CI: 12–24) for soap and 12 percentage points (95% CI: 5–19) for latrines. A greater proportion of people in intervention clusters reported washing their faces with soap (e.g. risk difference 21 percentage points; 95% CI: 15–27 for 0–5 year-old children) and using a latrine (e.g. risk difference 9 percentage points; 95% CI: 2–15 for 6–9 year-old children). Differences between the intervention and control arms were not statistically significant for many indicators until the programme had been implemented for at least a year; they did not decline during later study visits.

**Conclusion:**

The community- and school-based intervention was associated with improved hygiene access and behaviours, although changes in behaviour were slow and required several years of the intervention.

## Résumé

**Objectif:**

Déterminer si une intervention au niveau de l'eau, de l'assainissement et de l'hygiène pourrait avoir une influence sur les comportements en la matière, considérés comme importants dans la lutte contre le trachome.

**Méthodes:**

Nous avons mené un essai randomisé par grappes dans les régions rurales d'Éthiopie entre le 9 novembre 2015 et le 5 mars 2019. Nous avons réparti aléatoirement 20 échantillons où l'intervention consistait à développer les infrastructures d'assainissement et d'approvisionnement en eau et à promouvoir l'hygiène, et 20 échantillons n'ayant fait l'objet d'aucune intervention. Tous les échantillons du groupe d'intervention ont suivi une formation sur l'hygiène à l'école primaire, disposaient d'un point d'eau communautaire, d'un poste de lavage par ménage, de savon à domicile, et recevaient des visites de la part de travailleurs chargés d'enseigner les bonnes pratiques en matière d'hygiène. Nous avons évalué le niveau d'observance des mesures en effectuant des enquêtes annuelles au sein des foyers.

**Résultats:**

En l'espace de 3 ans, le nombre de postes de lavage, de savons et de latrines dans les ménages a davantage augmenté dans le groupe d'intervention que dans le groupe de contrôle: la différence de risque s'élevait à 47 points de pourcentage (intervalle de confiance de 95%, IC: 41–53) pour les postes de lavage, à 18 points de pourcentage (IC de 95%: 12–24) pour le savon et à 12 points de pourcentage (IC de 95%: 5–19) pour les latrines. La proportion de gens déclarant se laver le visage au savon était plus grande dans le groupe d'intervention (différence de risque de 21 points de pourcentage; IC de 95%: 15–27 pour les enfants de 0 à 5 ans), tout comme celle mentionnant l'usage de latrines (différence de risque de 9 points de pourcentage; IC de 95%: 2–15 pour les enfants de 6 à 9 ans). Pour de multiples indicateurs, il a fallu attendre minimum un an après l'instauration du programme pour que les variations observées entre les groupes d'intervention et de contrôle deviennent statistiquement significatives; ces variations se sont ensuite maintenues lors des visites ultérieures.

**Conclusion:**

Intervenir à l'école et au sein de la communauté a permis d'améliorer l'accès à l'hygiène et les comportements en la matière. Néanmoins, cette évolution prend du temps et plusieurs années d'intervention sont nécessaires.

## Resumen

**Objetivo:**

Determinar si una intervención de agua, saneamiento e higiene cambiaría las prácticas de higiene que se consideran importantes para el control del tracoma.

**Métodos:**

Se realizó un ensayo aleatorizado por grupos en zonas rurales de Etiopía del 9 de noviembre de 2015 al 5 de marzo de 2019. Se asignaron de manera aleatoria 20 grupos a una intervención que consistía en infraestructura de agua y saneamiento y promoción de la higiene, y 20 grupos a ninguna intervención. Todos los grupos de intervención recibieron un programa educativo sobre higiene en la escuela primaria, un punto de abastecimiento de agua en la comunidad, una estación de lavado en el hogar, jabón en el hogar y visitas a domicilio de profesionales dedicados a la promoción de la higiene. Se evaluó la fidelidad de la intervención mediante encuestas anuales en los hogares.

**Resultados:**

Durante los tres años, se observaron más estaciones de lavado, jabón y letrinas en los hogares de los grupos de intervención que en los de control: diferencia de riesgo de 47 puntos porcentuales (intervalo de confianza del 95 %, IC: entre 41 y 53) para las estaciones de lavado, 18 puntos porcentuales (IC del 95 %: entre 12 y 24) para el jabón y 12 puntos porcentuales (IC del 95 %: entre 5 y 19) para las letrinas. Un mayor porcentaje de personas en los grupos de intervención informó de que se lavaba la cara con jabón (p. ej., diferencia de riesgo de 21 puntos porcentuales; IC del 95 %: entre 15 y 27; para niños de 0 a 5 años) y de que usaba una letrina (p. ej., diferencia de riesgo de 9 puntos porcentuales; IC del 95 %: entre 2 y 15; para niños de 6 a 9 años). Las diferencias entre los grupos de intervención y de control no fueron significativas desde el punto de vista estadístico para muchos indicadores hasta que el programa se ejecutó durante al menos un año; tampoco disminuyeron durante las visitas posteriores del estudio.

**Conclusión:**

La intervención en la comunidad y en la escuela se asoció a una mejora del acceso y de las prácticas de higiene, aunque los cambios en las prácticas fueron lentos y necesitaron varios años de intervención.

## ملخص

**الغرض:**

تحديد ما إذا كان يمكن لتدخل المياه والصرف الصحي والنظافة أن يغير سلوكيات النظافة التي يعتقد أنها مهمة للسيطرة على التراكوما.

**الطريقة:**

قمنا بإجراء تجربة عشوائية جماعية في المناطق الريفية بإثيوبيا من 9 نوفمبر/تشرين ثاني 2015 إلى 5 مارس/آذار 2019. قمنا بالتوزيع العشوائي لعدد 20 مجموعة في أحد التدخلات يتألف من البنية التحتية للمياه والصرف الصحي وتعزيز النظافة، فضلاً عن 20 مجموعة دون تدخل. استلمت جميع مجموعات التدخل منهجًا للنظافة في المدارس الابتدائية، ونقطة مياه مجتمعية، ومحطة غسيل منزلية، وصابون منزلي وزيارات منزلية من عمال تعزيز النظافة. قمنا بتقييم دقة التدخل من خلال المسوحات المنزلية السنوية.

**النتائج:**

على مدى الثلاث سنوات، تم إنشاء المزيد من محطات الغسيل والصابون والمراحيض في المنازل في مجموعات التدخل مقارنة بمجموعات التحكم: نقاط اختلاف المخاطر 47 نقطة مئوية (فاصل الثقة 95%: 41 إلى 53) لمحطات الغسيل، 18 نقطة مئوية (فاصل الثقة 95%: 12 إلى 24) للصابون و12 نقطة مئوية (فاصل الثقة 95%: 5 إلى 19) للمراحيض. أبلغت نسبة أكبر من الأشخاص في مجموعات التدخل عن غسل وجوههم بالصابون (على سبيل المثال، اختلاف المخاطر 21 نقطة مئوية؛ فاصل الثقة 95%: 15 إلى 27؛ للأطفال من الولادة إلى عمر 5 سنوات) واستخدام مرحاض (على سبيل المثال، اختلاف المخاطر 9 نقاط مئوية؛ فاصل الثقة 95%: 2 إلى 15 للأطفال بعمر 6 إلى 9 سنوات). لم تكن الاختلافات بين ذراعي التدخل والتحكم ذات دلالة إحصائية بالنسبة للعديد من المؤشرات حتى تم تنفيذ البرنامج لمدة عام واحد على الأقل؛ ولم تنخفض خلال الزيارات الدراسية اللاحقة.

**الاستنتاج:**

ارتبط التدخل المعتمد على المجتمع والمدرسة بتحسين تحقيق النظافة والسلوكيات المحسّنة، على الرغم من أن التغييرات في السلوك كانت بطيئة وتطلبت العديد من سنوات من التدخل.

## 摘要

**目的:**

旨在确定针对水、环境卫生和个人卫生的干预措施是否可以改变普遍认为对沙眼控制起到重要作用的卫生行为。

**方法:**

我们于 2015 年 11 月 9 日至 2019 年 3 月 5 日在埃塞俄比亚农村地区进行了一项集群随机试验。我们随机将 20 个集群分配到包括针对水、环境卫生基础设施和个人卫生改善进行干预的干预组，将 20 个集群分配到未进行干预的对照组。所有干预组集群都接受了小学卫生课程、社区供水点、家庭洗手池、家用肥皂，以及来自卫生宣传工作者的家访。我们通过年度家庭调查评估干预的尽责程度。

**结果:**

在三年试验期间，干预组集群的家庭中出现洗手池、肥皂和厕所的几率比对照组更高：洗手池的风险差为 47 个百分点（95% 置信区间，CI: 41-53）；肥皂的风险差为 18 个百分点（95% 置信区间，CI: 12-24）；厕所的风险差为 12 个百分点（95% 置信区间，CI: 5-19）。较高比例的干预组集群人员报告称，他们使用肥皂洗脸（如 0-5 岁儿童的风险差为 21 个百分点，95% CI: 15-27），并且使用厕所（如 6-9 岁儿童的风险差为 9 个百分点，95% CI: 2-15）。在计划实施后至少一年时间，干预组和对照组之间的许多指标差异在统计上并不显著；这些指标在后来的研究随访中没有也下降。

**结论:**

以社区和学校为基础的干预与卫生条件的改善和行为的改变有关，尽管行为改变缓慢且需要多年的干预。

## Резюме

**Цель:**

Определить, могут ли меры в области водоснабжения, санитарии и гигиены изменить поведенческие навыки в области гигиены, которые считаются важными для контроля трахомы.

**Методы:**

Авторы провели кластерное рандомизированное исследование в сельской местности Эфиопии с 9 ноября 2015 г. по 5 марта 2019 г. 20 кластеров были выбраны случайным образом для внедрения меры, состоящей из пропаганды объектов инфраструктуры водоснабжения и санитарии и пропаганды гигиены, а также 20 кластеров, также случайно выбранных, были оставлены без внедрения каких-либо мер. Все экспериментальные кластеры были обеспечены программой по гигиеническим навыкам в начальной школе, коммунальными пунктами водоснабжения, бытовыми местами для мытья, хозяйственным мылом, а также к ним домой приходили работники по пропаганде гигиены. Авторы оценили правильность мер с помощью ежегодных опросов домохозяйств.

**Результаты:**

За 3 года в домохозяйствах экспериментальных кластеров было замечено больше мест для мытья, мыла и уборных, чем в контрольных кластерах: разница рисков составила 47 процентных пунктов (95%-й доверительный интервал, ДИ: 41–53) для мест для мытья, 18 процентных пунктов (95%-й ДИ: 12–24) для количества мыла и 12 процентных пунктов (95%-й ДИ: 5–19) для количества уборных. Большая часть людей в экспериментальных кластерах сообщили, что мыли лицо с мылом (например, разница рисков составила 21 процентный пункт; 95%-й ДИ: 15–27; для детей 0–5 лет) и пользовались уборной (например, разница рисков составила 9 процентных пунктов; 95%-й ДИ: 2–15; для детей 6–9 лет). Различия между экспериментальной и контрольной группами не были статистически значимыми по многим показателям до момента, когда после внедрения программы прошел по крайней мере год; они не снизились во время последующих визитов, предусмотренных исследованием.

**Вывод:**

Мера, внедряемая на уровне общества и школы, привела к улучшению доступа к объектам гигиены и поведенческих навыков в области гигиены, хотя изменения в поведении проявлялись медленно и потребовали нескольких лет применения этой меры.

## Introduction

The World Health Organization recommends improvements in water sources and promotion of facial cleanliness for trachoma elimination.^1^ However, very few randomized trials have assessed whether hygiene interventions targeted specifically for trachoma produce sustained changes in behaviour. Furthermore, the few existing trials have typically either not reported post-intervention facial hygiene behaviours or have been unable to show an effect.^2^^–^^10^

We report uptake of a hygiene intervention administered in the WASH Upgrades for Health in Amhara trial, a cluster-randomized trial in rural Ethiopia.^11^ A series of focus group discussions held before the trial showed variability in hygiene practices.^12^^,^^13^ Focus group participants reported that a main barrier to face-washing was the high cost of soap and that schoolchildren were key hygiene facilitators since they brought attitudes and perceptions gained in the classroom back home. With the results of these focus group discussions in mind, we developed a comprehensive water, sanitation and hygiene intervention to improve facial hygiene and latrine use behaviours. We used cluster-randomization since components of the intervention were administered at the community and school levels. The inclusion of a control group allowed us to estimate the true effect of the intervention.

The aim of our study was to determine whether a comprehensive water, sanitation and hygiene intervention could change hygiene behaviours. We hypothesized that the intervention would result in changes in water, sanitation and hygiene infrastructure and behaviour relative to control (i.e. non-intervention) communities, with changes persisting over the 3 years of the trial.

## Methods

### Design

The WASH Upgrades for Health in Amhara trial was a parallel-group, cluster-randomized trial conducted from 9 November 2015 to 5 March 2019. The trial protocol is reported elsewhere.^11^ Briefly, 20 clusters were randomized to a comprehensive water, sanitation and hygiene intervention and 20 control clusters were randomized to no intervention (clinicaltrials.gov NCT02754583). The control group will receive the same water, sanitation and hygiene intervention at the end of the trial. The primary pre-specified outcome of the trial was ocular chlamydia infection but we collected many indicators of intervention fidelity (i.e. the degree to which an intervention is implemented as intended) and adherence as intermediate outcomes (e.g. water and sanitation infrastructure uptake and self-reported hygiene-related behaviours), and these indicators are the subject of the present report. We obtained ethical approval for the study from: University of California, San Francisco, United States of America (USA); Emory University, Atlanta, USA; Ethiopian Ministry of Science and Technology; and Food, Medicine and Health Care Administration and Control Authority of Ethiopia. Because of high levels of illiteracy, we obtained verbal consent from all participants or their guardians before randomization and at all monitoring visits.

### Study setting

The trial took place in three districts of WagHemra Zone, Amhara Region, Ethiopia, an arid and mountainous area. Preliminary surveys documented hyperendemic trachoma and poor access to water and sanitation.^14^ A severe drought affected the study area in 2015–2016. A government health extension worker programme serves rural communities which includes, among other things, community-based hygiene education.^15^^,^^16^

### Eligibility

We defined randomization units by primary-school catchment area: all schoolchildren in the intervention clusters were eligible for the school-based interventions and all households within a 1.5 km radius of a pre-specified potential water point were eligible for community-based interventions.

### Census

We conducted a geohydrological survey before the study to identify the most suitable sites for construction of water points in each school catchment area. We conducted a census of all households within a 1.5 km radius of this potential water point before randomization and then each year until the end of the study.

### Randomization

After the baseline census was complete, the trial biostatistician randomized the study clusters in a 1:1 ratio to the water, sanitation and hygiene intervention arm or to the control arm, using R version 4 (R Foundation for Statistical Computing, Vienna, Austria). The study coordinator assigned the allocated intervention; allocation was concealed since it was done after the baseline census. We could not mask participants and data collectors due to the nature of the intervention, although we did not inform the data collectors of the randomization allocation.

### Intervention

The water, sanitation and hygiene intervention consisted of improvements in water and sanitation infrastructure and hygiene promotion, implemented in both community-based and school-based settings.^11^ We developed the study messaging and materials with input from government officials, school and community leaders, and community members, and organized our hypothesized causal pathway of desired hygiene behaviour changes in a logic model (i.e. a graphic representation of shared relationships).^17^ The intervention focused on two simple messages, repeated across different settings: (i) use soap and water to wash a child’s face twice a day; and (ii) always use a latrine for defecation. Each intervention cluster had a water point constructed at the pre-specified site. All households that had been counted in the census received a wash station (i.e. 25 L jerry can with a tap), a mirror, an illustrated 65-page educational hygiene book and a monthly supply of four bars of soap. We deployed salaried hygiene promotion workers to the study communities, where they lived and integrated themselves in community life. These workers made regular visits to each household, and spent time with the members of each household to better understand their attitudes, perceptions and motivations, and to help households identify their specific hygiene gaps and goals. The hygiene book had chapters dedicated to face-washing, clothes-washing, hand-washing, water collection, latrine use, latrine construction and wash-station construction. Each chapter had a self-assessment and ideas for specific steps that could be taken to improve the hygiene of the household and its members.^18^ Hygiene promotion workers reviewed a different chapter of the hygiene book at each visit. They finished the visit by asking an adult in the household to rate their own household on a water–sanitation–hygiene ladder and to set concrete goals on how to improve the level of hygiene at the household. Priests and community health volunteers (i.e. the government-promoted women’s health development army) received annual hygiene training and were asked to stress hygiene messages in their encounters with the public. We did not directly provide latrines because government policy discourages subsidization of latrines and instead asks households to contribute their own resources for latrine construction and maintenance.^19^ Nonetheless, hygiene promotion workers, priests and health volunteers encouraged latrine construction and use during their encounters with household members. School-based interventions targeted primary schools (i.e. children aged about 6–9 years) and included a primary-school hygiene curriculum, teaching aids and an instruction manual for extracurricular clubs dedicated to promoting hygiene at the school and in the community. The curriculum targeted hygiene behaviours considered important for reducing the risk of trachoma and enteric diseases. The curriculum consisted of five or six lessons per school grade for grades 1–4, with an emphasis on class participation (e.g. picture sorting, worksheets, drawing, role play, dramas and songs).^20^ Teachers and principals received training on the curriculum annually before the start of the school year.

The control clusters received none of these interventions during the study period, but existing government-supported hygiene programmes continued in both the intervention and control clusters.

### Assessment of fidelity

We considered intervention fidelity from two perspectives: first, the extent to which the components of the intervention were delivered as intended (i.e. adherence to intervention content, coverage, frequency and duration), and second, whether the study participants became engaged with the intervention and changed their hygiene behaviours (i.e. participant responsiveness).^21^^,^^22^ We took information on intervention delivery from study records generated during the course of implementing the intervention in the 20 clusters. We took information on participant responsiveness from an annual survey of a random 33% sample of households from all 40 communities. We administered this survey in both the intervention and control communities during the annual census at months 0, 12, 24 and 36.

### Statistical considerations

We modelled community-level summary statistics at all four study time points (months 0, 12, 24 and 36) in repeated measures regression models that included a time by treatment interaction term to allow for the possibility that the association between the intervention and the fidelity outcome depended on the duration of time since the intervention started. For age-stratified individual-level questions, we only included households with a member of the relevant age group. We estimated risk differences from the regression model as absolute percentage point differences at each time point and did a linear test for trend over time, assessed by orthogonal polynomials. We calculated the overall risk difference as the average over the three post-randomization time points. The number of clusters for the trial was based on the primary outcome – ocular chlamydia. We calculated that assuming 50 households per cluster, an intraclass correlation coefficient of 0.1 (based on a previous study),^23^ an α value of 0.05 and an affirmative response in 50% of households in the control group, then 20 intervention and 20 control communities gave an 80% power to detect a 15 percentage point difference between the two study arms for any question on the household survey.

## Results

### Baseline characteristics

**Fig. 1:**
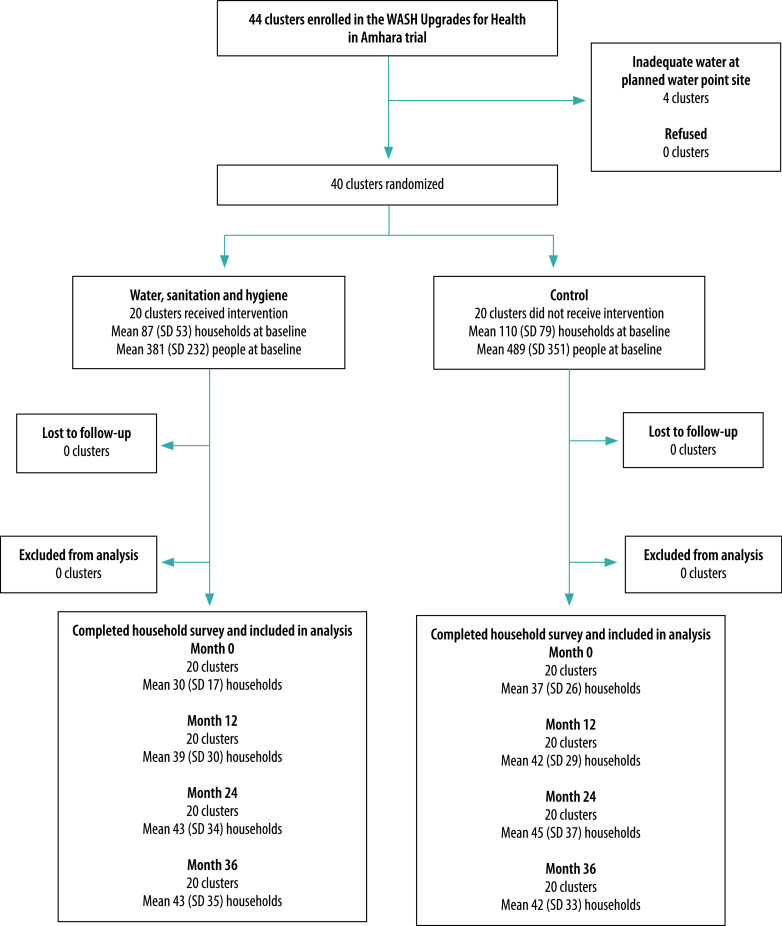
Flowchart of trial stages, and clusters and households included in the WASH Upgrades for Health in Amhara trial, Ethiopia, 9 November 2015 to 5 March 2019

Fig. 1 shows the flowchart of trial stages, and clusters and households included. There were no significant differences between baseline demographic characteristics of the intervention and control clusters (Table 1). We also found no substantive differences in water, sanitation and hygiene infrastructure or self-reported behaviours at the baseline household survey for the many indicators (data available in the data repository).^24^

SD: standard deviation.

**Table 1:** Baseline characteristics of study communities in the WASH Upgrades for Health in Amhara trial, Ethiopia, 2015

Characteristic	Mean (95% CI)	
	Intervention groups (*n* = 20)	Control groups (*n* = 20)
**Households, no.**	87 (63–112)	110 (74–147)
**Individuals, no.**	381 (273–490)	489 (325–654)
**Age in years, %**		
0–5	18.4 (17.2–19.7)	18.3 (16.9–19.7)
6–9	12.7 (11.8–13.6)	12.3 (11.7–12.9)
≥ 10	68.9 (67.3–70.4)	69.4 (67.8–71.0)
**Sex, %**		
Female	50.9 (49.5–52.2)	50.6 (49.5–51.8)
Male	49.1 (47.8–50.5)	49.4 (48.2–50.5)
**Distance from Sekota,^a^ km**	22.0 (16.4–27.7)	17.6 (12.9–22.3)
**Altitude, m**	2327 (2153–2501)	2289 (2119–2459)
**Household language, %**		
Amharic	66.4 (45.0–87.8)	56.5 (35.0–78.1)
Himtana (Agewgna)	33.6 (12.2–55.0)	43.2 (21.7–64.6)
Other	0.0 (0.0–0.0)	0.3 (0–0.8)
**Households with mobile telephone, %**	2.3 (0.2–4.3)	2.3 (0.8–3.8)

CI: confidence interval.

**^a^** The capital town of the WagHemra Zone and the largest urban area in the study area.

### Intervention delivery

We reviewed study records to assess the extent to which the delivery of the intervention adhered to the intended content, coverage, dose and frequency.

#### Water points

We arranged construction of water points in all 20 intervention communities during a severe drought (median time from randomization to construction was 5 months, interquartile range 3–7 months, range 1–15 months). Three types of water points were constructed depending on the local geohydrologic characteristics, including 13 spring developments, four hand-dug wells and three shallow boreholes. At some point during the study, 11 communities had an interruption in water-point functioning; all water points were subsequently repaired. Out of a possible maximum of 34 months, the cumulative period the water points were functional ranged from 15 to 33 months; median 25 months and interquartile range 21 to 29 months (data repository).^24^

#### Hygiene promotion workers

We trained nine hygiene promotion workers who went to the intervention communities about 3 months after randomization. We added another hygiene promotion worker at month 12 and two more at month 24. The hygiene promotion workers covered one to three clusters depending on community size and location. According to the records of the hygiene promotion workers, most households were visited at least six times a year (data repository).^24^

#### Water and sanitation hardware

According to study records, all new households counted at each census in the 20 intervention communities received wash stations and hygiene books as did all households identified by hygiene promotion workers as needing a replacement. Soap deliveries were delayed due to logistical issues. Households received soap each month starting immediately after the month-12 household survey, with study records documenting monthly soap distributions 10 times during the second year of the study and 12 times during the third year (data repository).^24^

#### Schools

According to annual interviews with school principals, all the schools received the curriculum for grades 1–4 over the study period, with only one school failing to implement the intervention during the first year. School water, sanitation and hygiene clubs were active in 18 intervention clusters during each of the three school years of the study (data repository).^24^

### Participant responsiveness

**Fig. 2:**
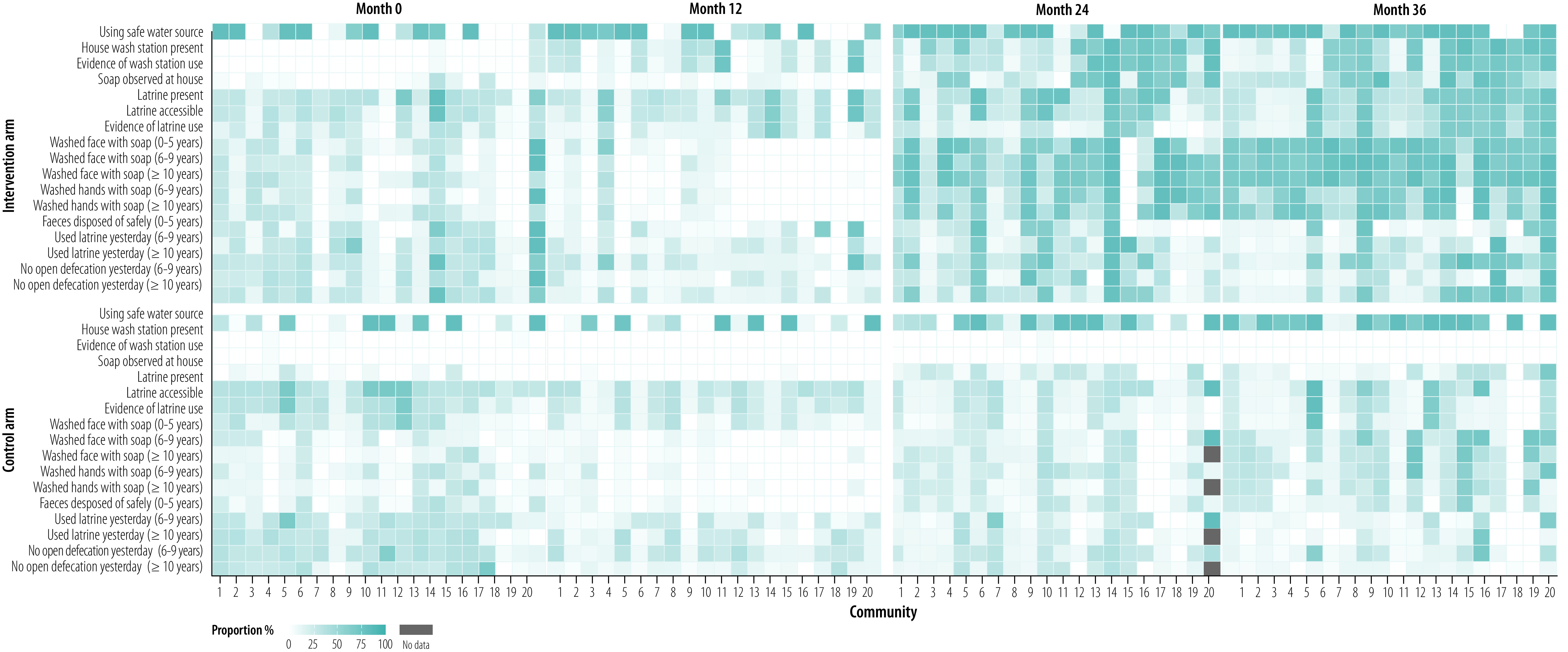
Proportion of households per community fulfilling selected water, sanitation and hygiene indicators at annual monitoring visits, Ethiopia, 9 November 2015 to 5 March 2019

Notes: The water, sanitation and hygiene interventions were rolled out in 20 communities (top panels), mostly between baseline (i.e. month 0) and month 12. No specific interventions were implemented in the control arm (bottom panels) during the study period. Each box shows the percentage of households or people per community with a positive response, with darker blue indicating higher levels. Numerators and denominators for each percentage are available in the data repository.^25^

On average, between 30 and 45 households per community in both the intervention and control arms completed a survey at each study visit (Fig. 1). A heat map of key household survey outcomes showed greater uptake of desired infrastructure and hygiene behaviours in the intervention arm (Fig. 2; available at https://www.who.int/publications/journals/bulletin/). 

#### Sanitation infrastructure 

**Fig. 3:**
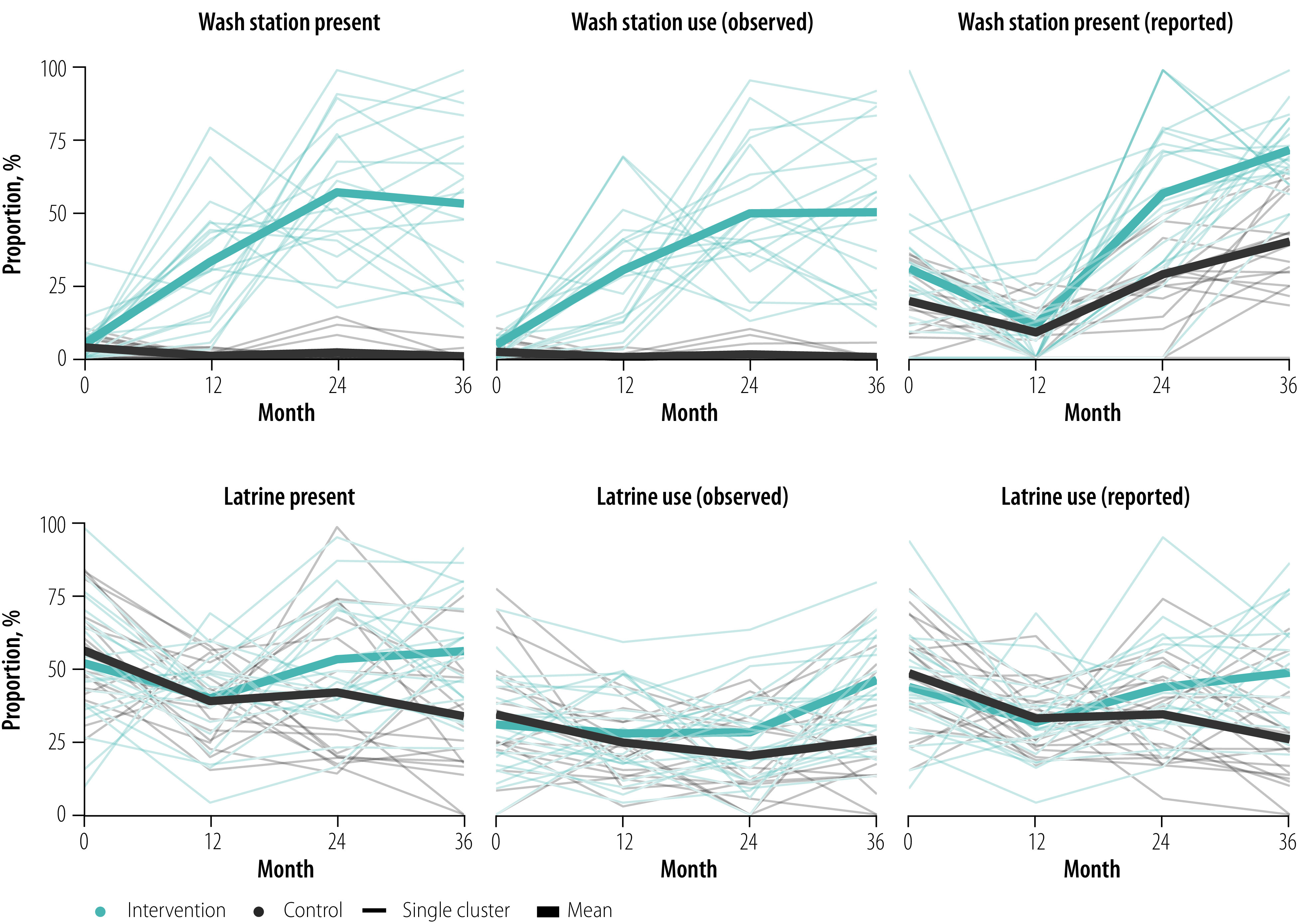
Trends in key hygiene indicators in WASH Upgrades for Health in Amhara trial, Ethiopia, 9 November 2015 to 5 March 2019

Notes: The presence and evidence of use of wash stations and latrines were based on observation by field workers who were not informed of the randomization allocation. Wash-station use was defined as the presence of water in the wash station container or wet ground surrounding the wash station. Latrine use was defined as the presence of flies, smell or faeces or a wet floor in the latrine. Caregiver-reported face-washing with soap in the past day is reported for children aged 0–5 years and self-reported latrine use in the past day is reported for individuals aged ≥ 10 years. Numerators and denominators for each percentage are available in the data repository.^25^

**Fig. 4:**
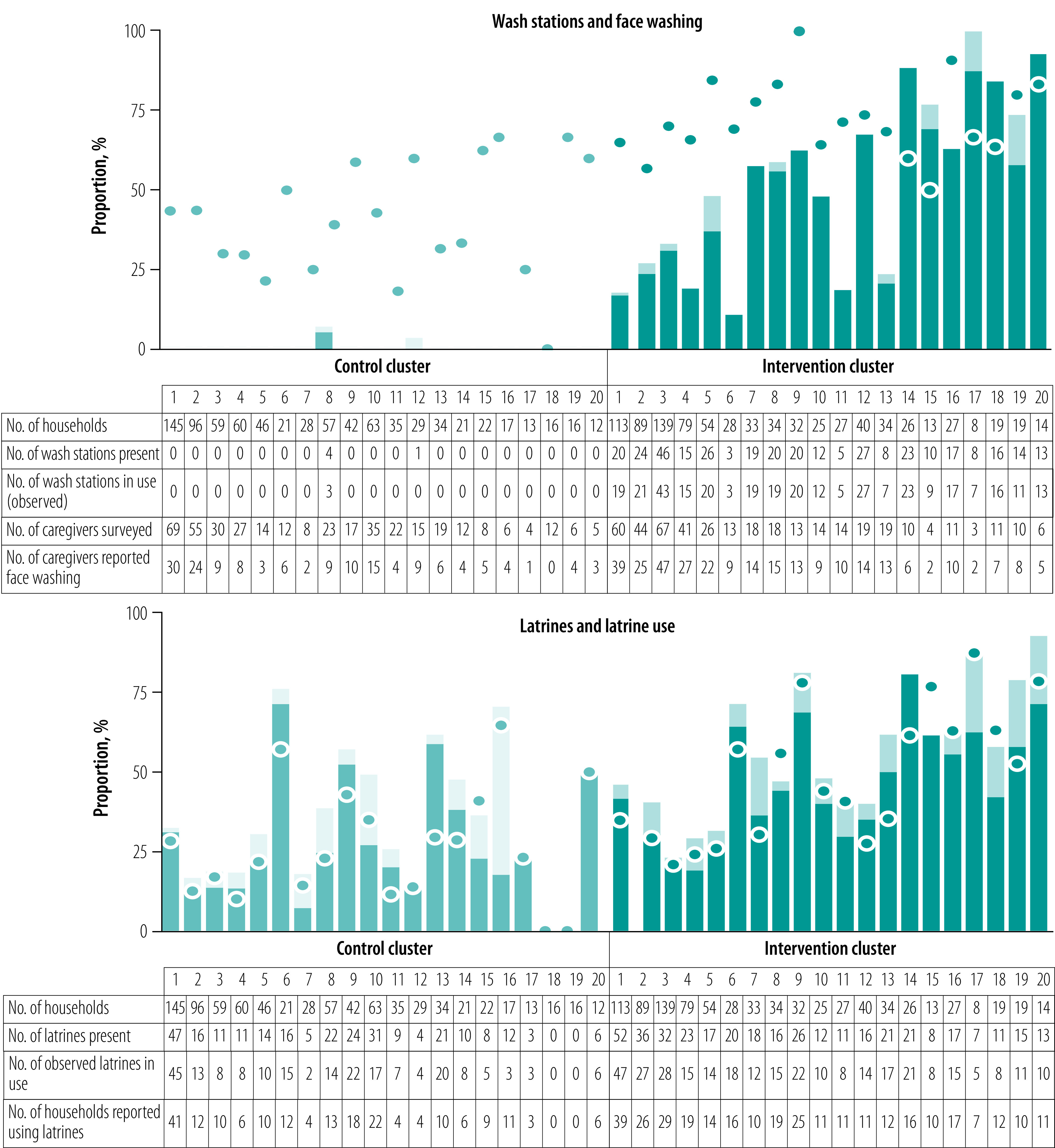
Key hygiene indicators in WASH Upgrades for Health in Amhara trial at end-point, Ethiopia, 2019

Notes: The stacked bars represent the observed presence (total height of the bar) and use (darker portion of the bar) of household wash station or latrine in each cluster. The dots represent caregiver-reported face-washing among children aged 0–5 years or self-reported latrine use among individuals aged ≥ 10 years.

**Fig. 5:**
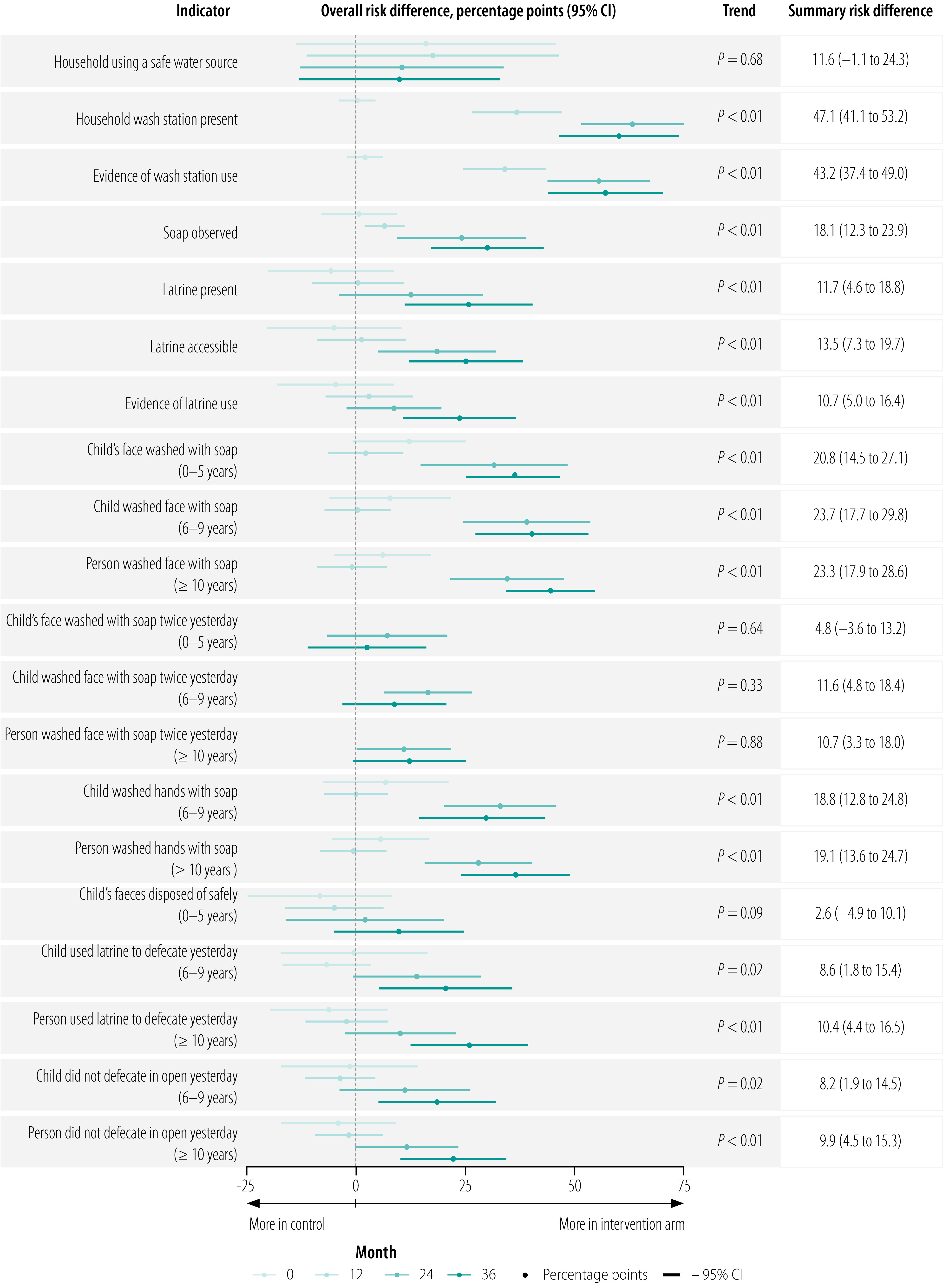
Risk difference in hygiene indicators between intervention and control arms of the WASH Upgrades for Health in Amhara trial, Ethiopia, 9 November 2015 to 5 March 2019

At month 36, latrine coverage was greater in the intervention clusters than control clusters; overall estimates were 57% (95% CI: 48–66%) in the intervention arm and 34% (95% CI: 24–44%) in the control arm (Fig. 3 and Fig. 4). Latrine coverage became significantly higher in the intervention arm than the control arm over the course of the study (Fig. 5) – overall risk difference 12 percentage points (95% CI: 5–19). 

CI: confidence interval.

Notes: For each question, the risk difference, calculated as percentage points, between the intervention and control arms is shown separately for each annual study visit. Risk differences greater than 0 show that the indicator was more common in the intervention arm than the control arm. A missing plot indicates the question was not asked at the study visit. The overall risk difference and 95% CI between the two arms across the three post-randomization study visits are shown to the right of the plot. The *P*-value tests for a linear trend across all study visits.

#### Wash station infrastructure

The intervention communities had an increase in wash stations and soap compared with control communities starting at the month-12 survey (Fig. 3, Fig. 4 and Fig. 5). By month 36, 54% (95% CI: 42–65%) of households in the intervention arm had wash stations compared with < 1% (95% CI: 0–1%) in the control arm. In addition, 53% (95% CI: 46–61%) of households in the intervention arm had soap compared with 27% (95% CI: 19–35%) in the control arm. Over the three post-randomization visits, the intervention arm had 47 (95% CI: 41–53) percentage points greater coverage of wash stations and 18 (95% CI: 12–24) percentage points greater coverage of soap than the control arm.

#### Household water use

At month 36, the proportion of households with evidence of wash station use (i.e. water present in or on the ground surrounding the wash container) was higher for the intervention communities (51%, 95% CI: 39–62%) than control communities (< 1%, 95% CI: 0–1%). Overall, wash station use was 43 (95% CI: 37–49) percentage points higher in the intervention communities over the three post-randomization visits than the control communities. We found no difference in the total volume of water collected or frequency of clothes-washing between the two arms over the course of the study (data repository).^24^

#### Washing behaviours

Face-washing with soap was more common in the intervention arm across all age groups starting at month 24 (Fig. 5). By month 36, the proportion of households with a child 5 years or younger in which water and soap were used to wash the child’s face the previous day was 72% (95% CI: 67–77%) in the intervention arm compared with 40% (95% CI: 33–48%) in the control arm. Across all post-intervention visits, face-washing with soap among children  5 years or younger was 21 (95% CI: 15–27) percentage points higher in the intervention arm than the control arm.

#### Latrine behaviours

Self-reported latrine use was more common and self-reported open defecation less common in the intervention arm than the control arm across all age groups starting at the month-24 survey (Fig. 5). For example, at month 36, the percentage of households with a 6–9-year-old child in which the child used a latrine the previous day was 36% (95% CI: 24–47%) in the intervention arm and 17% (95% CI: 11–24%) in the control arm. Across all post-intervention visits, latrine use in this age group was 9 (95% CI: 2–15) percentage points higher in the intervention arm than the control arm. Estimates of self-reported open defecation the previous day provided similar conclusions, as did analyses of the other age groups (Fig. 5).

## Discussion

We document the successful delivery and uptake of a comprehensive and intensive water, sanitation and hygiene intervention in rural Ethiopia that was based on previous formative research and conducted with local input and collaboration. Annual hygiene surveys found significant increases in self-reported face-washing with soap and latrine use in the intervention communities across all ages. Behaviour changes in the intervention arm generally became most evident at the 24-month monitoring visit and showed no evidence of declining by the final visit. Taken together, these data support the ability of a sustained, intensive water, sanitation and hygiene programme to change hygiene behaviours in a rural African setting.

Sustained behaviour change is difficult to achieve.^26^ Identifying causes of an existing behaviour, as well as the barriers to and facilitators for changing the behaviour, is thought to be important when designing interventions to address a specific behaviour given that behaviours vary by sociocultural context and are subject to a variety of direct and indirect influences.^26^^,^^27^ For the present study, formative research was essential to better understand the factors contributing to face-washing and defecation behaviours in the study area. The considerable variability in attitudes between communities led us to make use of hygiene promotion workers to adapt the hygiene messages to the specific contexts, with the purpose of helping households identify hygiene gaps and goals themselves. Hygiene promotion workers lived in the communities they served, helping to make them trusted community members and advisers.

By the end of the study, face-washing with soap had become much more common in the intervention clusters than the control clusters, an encouraging sign since soap has been found to play a key role in clearing ocular discharge and ocular chlamydia.^28^ Practise of the main hygiene message – face-washing with soap twice a day – was also more common in intervention clusters, although this practice was reported by only 26–43% of participants by month 36. These results, while affirming the challenges inherent in behavioural interventions, nonetheless point to positive changes in face-washing during the study period.

The estimated latrine coverage in the intervention communities was only 57% at the final study visit and estimated latrine use was even lower (e.g. 36% among children aged 6–9 years and 49% among individuals ≥10 years). These figures are lower than the 80% target that has been found to be associated with reductions in trachoma in observational studies.^29^ Government policy did not allow subsidization of latrines for the present study and thus the improvements in the intervention arm derive only from the latrine-promotion messaging. Despite the lack of subsidies, latrine coverage and use were about 20 percentage points higher in the intervention clusters than the control clusters by the final study visit. These findings are in line with the results of a meta-analysis that found sanitation interventions produced a mean increase of 14 percentage points in latrine coverage relative to a control arm.^30^ Some studies have achieved higher sanitation coverage, although in these studies latrine construction was subsidized.^31^^–^^34^ Overall, our results suggest that although latrine uptake was gradual and latrine use was not universal, sanitation promotion by community-based hygiene promotion workers and a school curriculum were capable of producing incremental change in hygiene behaviour.

The two most important behaviours targeted by the intervention were latrine use and face-washing with soap. Of these, the intervention had a stronger influence on self-reported face-washing behaviour. Although there are many possible reasons for a greater effect on face-washing, it is noteworthy that wash stations and soap were given to the intervention clusters, thus providing the necessary infrastructure to encourage the behaviour change. In contrast, latrine infrastructure was not provided or subsidized. Health programmes may thus maximize changes in hygiene behaviour by facilitating access to water, sanitation and hygiene infrastructure, which removes a substantial barrier for many people in poor rural settings.

Our study has several strengths. The intervention was based on formative research done locally. Randomization provided confidence that the behaviour changes were as a result of the intervention, rather than the result of a time trend. Household survey responses were representative of the general population given the population-based sampling. The surveys took place at the same time each year, thus minimizing seasonal variability in responses. The intervention was continuously implemented for a relatively long time, which gave time for behaviours to gradually change.

Limitations of our study include the reliance on self-reported behavioural data. Respondents may have overstated their hygiene behaviours, and if this overstatement was different between the control and intervention arms, this could have biased the trial. Although we did not inform data collectors of the randomization allocation, the study could not be masked and thus was subject to the possibility of different co-interventions between the two arms. The study was designed to assess the intervention’s efficacy under ideal conditions, and not its effectiveness in the real world. The scope, intensity and cost of the intervention, while feasible for a research programme, might be difficult for a health programme to administer. A 3-year implementation period may be too short to observe the full effects of a behaviour-change intervention. Finally, the generalizability of the findings outside this part of Ethiopia is unclear, especially given the importance of local government policies as well as local customs on hygiene practices.

In summary, we found that while participant engagement was not universally high across all intervention communities, an intensive water, sanitation and hygiene intervention nonetheless produced positive behaviour changes. Evidence for these changes only emerged after 2 years of the intervention, highlighting the importance of long-term programmes for hygiene improvements.
